# Synthesis of photoresponsive and photoemissive ultrathin 2D nanosheets of In_2_S_3_ achieved through a new single source molecular precursor†

**DOI:** 10.1039/d2ra05000e

**Published:** 2022-09-27

**Authors:** Gourab Karmakar, Adish Tyagi, Alpa Y. Shah, Liladhar B. Kumbhare, A. P. Wadawale, G. Kedarnath, Vishal Singh

**Affiliations:** Chemistry Division, Bhabha Atomic Research Centre Mumbai 400 085 India tyagia@barc.gov.in kedar@barc.gov.in; Homi Bhabha National Institute Anushaktinagar Mumbai 400 094 India; Materials Science Division, Bhabha Atomic Research Centre Mumbai 400 085 India

## Abstract

Indium sulfide, a two-dimensional semiconductor material, has emerged as a promising candidate for cost-effective and sustainable solar cells. This report deals with the facile preparation of colloidal In_2_S_3_ with a new ultrathin nanosheet (NS) morphology. The synthesis was mediated through a new structurally characterized single source molecular precursor. The crystal structure, phase purity, and morphology of the NSs were thoroughly investigated by pXRD, Raman, XPS, and electron microscopic techniques. AFM studies revealed that the NSs have an average thickness of ∼1.76 nm. The optical studies confirm quantum confinement in the as-prepared NSs with a blue shift in the direct band gap, which lies in the optimal range suitable for solar cell application. Furthermore, photoluminescence studies indicate strong emission by these NSs in the blue region. The as-synthesized In_2_S_3_ NSs-based prototype photoelectrochemical cell exhibit high photostability and photoresponsivity, which make them suitable candidates for sustainable solar cells.

## Abstract 2

Quantum confined ultrathin nanosheets of In_2_S_3_ were synthesized from a new structurally characterized molecular precursor. The prototype photoelectrochemical cell based on the material exhibited high photostability and photoresponsivity.

## Graphical abstract

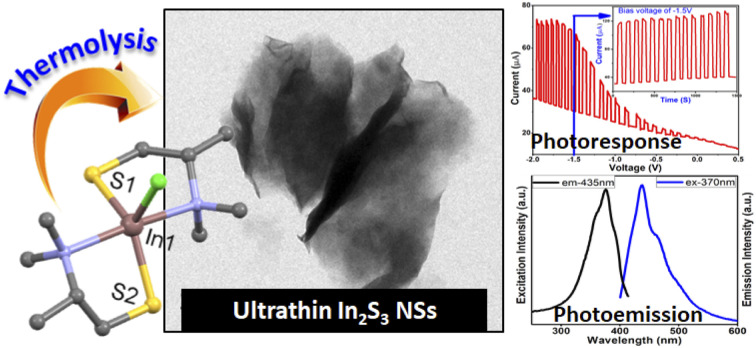

## Introduction

The research on two-dimensional (2D) materials has witnessed a tremendous boost in recent times owing to their intriguing physicochemical properties, such as enhanced optical transparency, excellent conductivity, and outstanding mechanical strength.^1–5^ Among them, layered metal chalcogenide (LMCs) semiconductors have garnered unprecedented attention and have been explored widely in order to complement graphene due to their promising optoelectronic properties.^6^ These materials in ultrathin dimension (nanosheets; NSs) are considered to be quite exquisite due to their distinctive electronic, physical, and structural properties.^7,8^ For example, charge carriers exhibit large mobility in these materials. The conductivity of these materials is highly sensitive to electrostatic perturbation *via* photogenerated carriers adjacent to the surface layer. This makes 2D materials quite sophisticated for extraordinary gain photodetection due to the photogating effect.^9^ Moreover, ultrathin LMC NSs with thickness (*t*) in the quantum confinement regime (*t* ≤ exciton Bohr radius) are of particular interest as they blend the extraordinary properties of 2D nanomaterials with the advantages of solution processability.^10^ There are certain 2D LMCs, especially those consisting of post transition metal elements (InSe, In_2_Se_3_, In_2_S_3_) that exhibit strong interaction with light, leading to their application in ultrathin and flexible optoelectronics with high-power-density.^11^ Indium sulfides, in nanoregime, exhibit many unique optoelectrical and thermal properties, coupled with high charge carrier concentration, which make them potential materials for energy and societal applications such as in solar cells,^12^ photocatalysis,^13^ photodetectors,^14^ and bioimaging.^15^ In a recent study, In_2_S_3_ quantum dots (QDs) produced from InCl_3_ and Na_2_S was successfully applied in a UV photovoltaic detector.^16^ To date, colloidal NSs of a handful of semiconductor materials have been successfully synthesized, *viz.*, CdX (X = S, Se, Te), SnX (X = S, Se), Cu_2−*x*_X (X = S, Se), PbS, InSe, and In_2_Se_3_.^17^ However, reports on NSs of indium sulfide are rather rare. In addition, producing high quality indium sulfide NSs is often limited by the inherent drawbacks of conventional synthetic procedures such as hydrothermal, template, microwave, and solvothermal approaches using multiple precursors.^18^ Single source molecular precursor (SSP) route is an alternative low-cost pathway to achieve these materials, which often proves to be advantageous because it provides better control over the stoichiometry and leads to materials with better phase purity and lower defect concentration.^19^ Conventional SSPs for indium sulfide materials include indium thiolates, thiocarboxylates, dithiocarbamates, dithioimidodiphosphinates, and dithiobiurets.^20–22^ Nonetheless, all these strategies to synthesize In_2_S_3_ have always yielded either large nanoparticles and in very few instances afforded quantum dots (QDs). This leaves an opportunity to explore the 2D morphology of In_2_S_3_ nanostructures, of which hardly any reports can be found in the literature.

In this study, we have designed and synthesized an air- and moisture-stable heteroleptic indium complex with an internally-functionalized hemilabile aminoalkylthiolate ligand. This structurally characterized SSP undergoes rapid low-temperature thermolysis in oleylamine (OAm) to produce ultrathin NSs of phase pure cubic In_2_S_3_. These NSs were characterized by pXRD, Raman, and XPS analysis, whereas the morphology and thickness of the NSs were determined by SEM, TEM, and AFM techniques. The optical properties were explored by DRS, UV-vis, and photoluminescence spectroscopy. We believe that the precursor-mediated synthesis of this new morphology of In_2_S_3_, accompanied by quantum confinement, will contribute to the widening of the research horizon of molecular precursors for optoelectronic materials with tailor-made morphologies and optical properties.

## Experimental

### Materials and methods

InCl_3_, oleylamine (OAm), and analytical grade solvents were procured from commercial sources. The dimer of the ligand (2-[(dimethylamino)propyl]-1-sulfide; Me_2_NCH(Me)CH_2_S^−^): bis(2-[(dimethylamino)propyl]-1-sulfide); [Me_2_NCH(Me)CH_2_S]_2_ [^1^H NMR (CDCl_3_): *δ* 1.00 (d, CH*Me*), 2.19 (s, N*Me*_2_), 2.52 (m, C*H*_2_S), 2.77–2.92 (m, NC*H*) ppm. ^13^C{^1^H} (CDCl_3_): *δ* 12.7 (s, CH*Me*), 40.0 (s, N*Me*_2_), 42.4 (s, N*C*H), 58.2 (*C*H_2_S) ppm] was prepared according to the literature method.^23^ Elemental analyses were carried out on a Thermo Fisher Flash EA-1112 CHNS analyzer. The ^1^H and ^13^C{^1^H} NMR spectra were recorded on a Bruker Advance-II NMR spectrometer operating at 300 and 75.47 MHz, respectively. Chemical shifts are relative to the internal chloroform peak for ^1^H and ^13^C{^1^H} NMR spectra.

Thermogravimetric analyses (TGA) were carried out on a Nitzsch STA 409 PC-Luxx TG-DTA instrument, which was calibrated with CaC_2_O_4_·H_2_O. The TG curves were recorded at a heating rate of 10 °C min^−1^ under the flow of argon. X-ray powder diffraction patterns were obtained on a Philips PW-1820 diffractometer using Cu-K_α_ radiation. XPS measurements were carried out using Mg-K_α_ (1253.6 eV) source and DESA-150 electron analyzer (Staib Instruments, Germany). For XPS analysis, a film was prepared by drop coating the sample on the glass substrate and drying under an IR lamp. The binding energy scale in XPS was calibrated to the C 1s line at 284.5 eV. All the deconvolutions and fittings were done by the CasaXPS software. Optical diffuse reflectance measurements in the range of 200–1100 nm (1.12–6.2 eV) were performed on a JASCO V-670 two-beam spectrometer with a diffuse reflectance (DR) attachment consisting of an integration sphere coated with BaSO_4_, which was used as a reference material. The measured reflectance data were converted to absorption (*A*) using the Kubelka–Munk remission function.^24^ The band gaps of the samples were estimated by extrapolating the linear portion of the plot to *X* (energy) axis. SEM and EDS measurements were carried out on an ULTRA 55 FESEM of Zeiss and Oxford Inca instruments, respectively. A Zeiss Libra 200 FE Transmission electron microscope (TEM) operating at an acceleration voltage of 200 kV was used for TEM studies. The samples for TEM and SAED were prepared by placing a drop of the sample dispersed in acetone/toluene on a carbon-coated copper grid.

The intensity data for In[Me_2_NCH(Me)CH_2_S]_2_Cl (1) was collected from a single crystal at 298(2) K on a XtaLAB Synergy, Dualflex, HyPix four-circle diffractometer with a micro-focus sealed X-ray tube using a mirror as a monochromator and a HyPix detector. The diffractometer was equipped with a low-temperature device and used Cu-K_α_ radiation (*λ* = 1.54184 Å). The unit cell parameters (Table 1) were determined from 25 reflections measured by a random search routine. All data were integrated with CrysAlis PRO and a multi-scan absorption correction using SCALE3 ABSPACK was applied.^25^ The structures were solved by iterative methods using OLEX 1.2 and refined by full-matrix least-squares methods against *F*^2^ using SHELXL-2017/1.^26^ All non-hydrogen atoms were refined with anisotropic displacement parameters. The hydrogen atoms were refined isotropically on the calculated positions using a riding model with their *U*_iso_ values constrained to 1.5 times the *U*_eq_ of their pivot atoms for terminal sp^3^ carbon atoms and 1.2 times for all other carbon atoms. Disordered moieties were refined using bond length restraints and displacement parameter restraints. Molecular structures were drawn using ORTEP.^27^

**Table 1:** Crystallographic and structural determination data for In[Me_2_NCH(Me)CH_2_S]_2_Cl (1)

		In[Me_2_NCH(Me)CH_2_S]_2_Cl (1)
Chemical formula		C_10_H_24_ClInN_2_S_2_
Formula weight		386.70
Crystal size/mm^3^		0.10 × 0.05 × 0.05
Crystal system/space group		Monoclinic/*P*2_1_/*c*
Unit cell dimensions	*a*/Å	10.6094(6)
	*b*/Å	12.8828(7)
	*c*/Å	11.8236(6)
	*α*	90
	*β*	98.915(4)
	*γ*	90
	Volume/Å^3^	1596.51(15)
	*Z*	4
	*D*_c_/g cm^−3^	1.609
	*μ*/mm^−1^	15.644
	*F*(000)	784
Limiting indices		−13 ≤ *h* ≤ 13; −16 ≤ *k* ≤ 15; −14 ≤ *l* ≤ 14
*θ* range of data collection/°		4.218–77.580
No. of reflections collected/unique		3209/2511
No. of data/restraints/parameters		3209/0/151
Final *R*_1_, ω*R*_2_ indices [*I* > 2*σ*(*I*)]		0.0594, 0.1520
*R*_1_, ω*R*_2_ (all data)		0.0724, 0.1603
Goodness of fit on *F*^2^		1.032
Largest diff. peak and hole (e Å^−3^)		1.324 and −0.985

### Synthesis of In[Me_2_NCH(Me)CH_2_S]_2_Cl (1)

To a freshly prepared solution of Me_2_NCH(Me)CH_2_S^−^Na^+^ [*in situ* obtained by reducing [Me_2_NCH(Me)CH_2_S]_2_ (300 mg, 1.26 mmol) and NaBH_4_ (95.3 mg, 2.52 mmol) in a toluene–methanol solution], solid InCl_3_ (278.6 mg, 1.26 mmol) was added, and the reaction mixture was stirred at room temperature for 3 h. The solvents were evaporated under vacuum and the residue was washed thoroughly with diethyl ether, followed by water to remove sodium chloride. The final product was dried under reduced pressure and recrystallized from hot methanol to afford colorless crystals (yield: 426.7 mg, 88%), mp 216 °C (dec.). Anal. calcd for C_18_H_21_InN_6_Se_3_: C, 31.05; H, 6.25; N, 7.24; S, 16.58%. Observed: C, 30.97; H, 6.22; N, 7.12; S, 16.29%. ^1^H NMR (CDCl_3_): *δ* 1.21 (d, CH*Me*), 2.72 (s, N*Me*_2_), 2.56 (m, C*H*_2_S), 3.23 (m, NC*H*) ppm. ^13^C{^1^H} (CDCl_3_): *δ* 12.5 (s, CH*Me*), 41.2 (s, N*Me*_2_), 42.7 (s, N*C*H), 59.8 (*C*H_2_S) ppm.

### Preparation of indium sulfide nanostructures

The indium sulfide nanostructures were prepared using In[Me_2_NCH(Me)CH_2_S]_2_Cl (1) as a molecular precursor by the hot-injection method employing OAm as a high boiling solvent. In a typical hot injection method, 9 mL OAm was taken in a three-necked round bottom flask and degassed at 110 °C under nitrogen flow for 30 min. Subsequently, the temperature was raised to 150 °C. In the preheated solvent, the required amount of precursor 1 (200 mg, 0.52 mmol) dispersed in OAm (1 mL) was rapidly injected. The reaction temperature dropped by about 20 °C upon injection; however, the set temperature was attained rapidly. The temperature was maintained for 1 min, after which the heat source was removed and the reaction mixture was allowed to cool to 60 °C, followed by the addition of methanol to ensure the complete precipitation of nanostructures. The synthesized orange colored material was collected after repeated washing with methanol and toluene mixture, followed by centrifugation to remove excess capping agent.

### Photoelectrochemical cell experiment

The photoresponsiveness of the nanostructures was measured in a photoelectrochemical cell prepared using silicon/In_2_S_3_ geometry as the working electrode, Pt wire as the counter and pseudo reference electrodes, and Na_2_S (0.6 M) : Na_2_SO_3_ (0.8 M) (1 : 2) as the electrolyte. The working electrodes of silicon/In_2_S_3_ were prepared by drop casting a colloidal solution of In_2_S_3_ nanostructures in chloroform on the rough surface of n-type Si wafer of (100) orientation having a thickness of 200 μm and resistivity of 1–10 Ω cm. The drop casted film was dried at 140 °C for 2 h. The process was repeated many times till a uniform coating was obtained on the silicon surface. A fluorescent white lamp (36 W, UV content < 3%) was used as the radiation source with the light intensity at the cell being 200 μW cm^−2^.

## Results and discussion

**Fig. 1:**
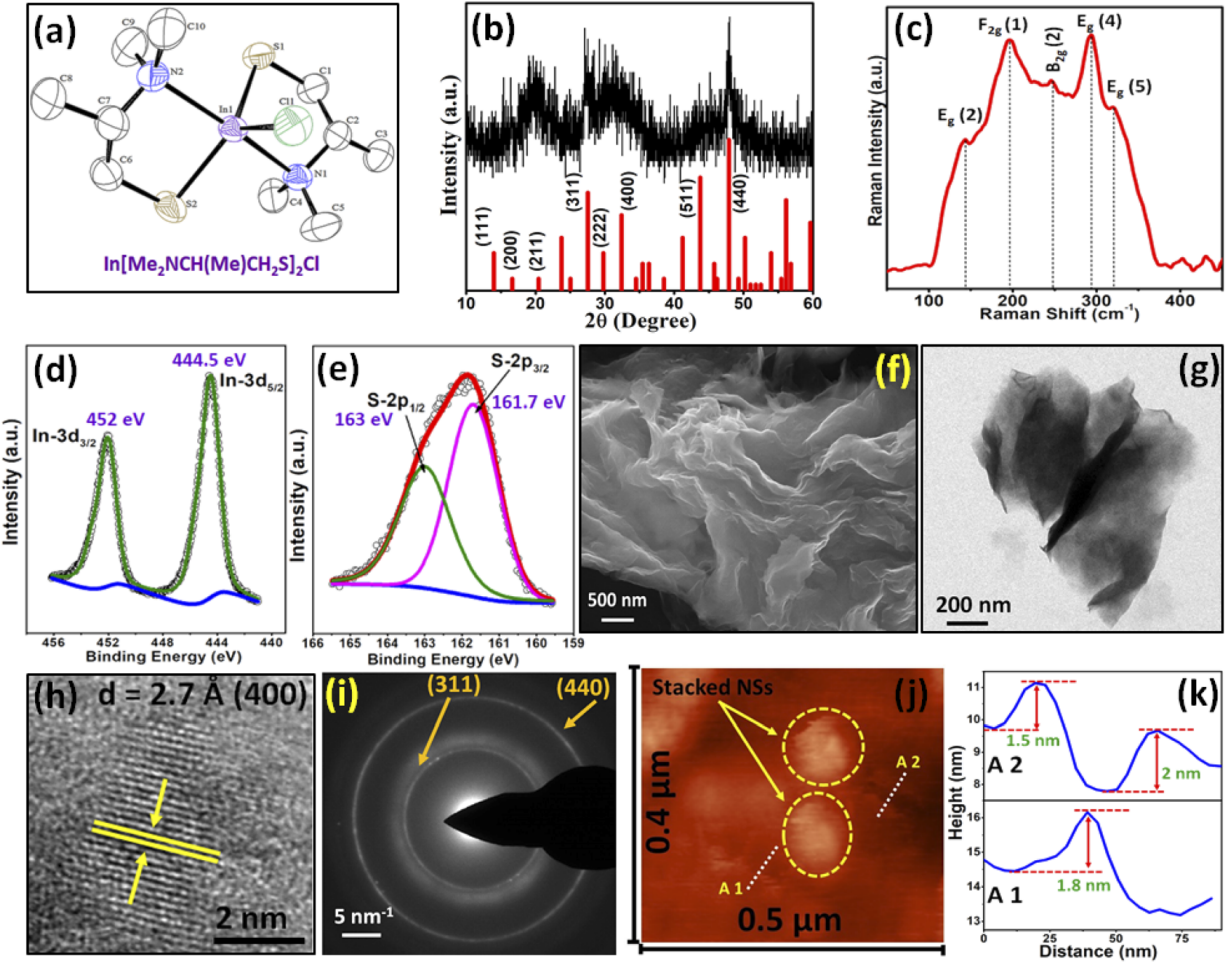
(a) Molecular structure of In[Me_2_NCH(Me)CH_2_S]_2_Cl (1), (b) XRD pattern of indium sulfide nanostructures synthesized by thermolysis of 1 in OAm at 150 °C for 1 min (cubic In_2_S_3_; JCPDS 32-0456), (c) Raman spectra, (d) XPS In 3d_3/2_ and In 3d_5/2_, and (e) S 2p spectrum, (f) SEM, (g) TEM, (h) HRTEM, (i) SAED, (j) AFM image, and (k) height-profile analysis of In_2_S_3_ NSs.

The SSP, In[Me_2_NCH(Me)CH_2_S]_2_Cl (1), was synthesized at room temperature and initially characterized by elemental analysis and NMR spectroscopy. It is worth mentioning that any attempt to isolate the homoleptic complex of indium with the ligand always resulted in an insoluble polymeric compound, for which, complete structural characterization is burdensome. This is conceivable from the fact that the complexation of heavier metals with thiolate ligands was often found to afford insoluble polymers.^28^ Thus, a heteroleptic approach was adopted with the substitution of only two –Cl atoms on indium with the ligand. The expected resonances and peak multiplicities in the ^1^H and ^13^C{^1^H} NMR spectra coupled with elemental analysis confirm the purity of the complex. Colorless diffraction quality single crystals of 1 were grown by recrystallization from hot methanol. The molecular structure of 1 with the atomic numbering scheme is shown in Fig. 1a. The crystallographic and structural determination data along with the refinement parameters are given in Table 1, and the selected interatomic parameters are given in Table 2. The complex structure features distorted trigonal bi-pyramidal geometry around the central indium atom. The N1 and N2 of the two chelating Me_2_NCH(Me)CH_2_S^−^ ligands are in axial positions, while the equatorial positions are occupied by S1 and S2 of the two ligands and the Cl atom. The ligands chelate the indium to form two five-membered metallacycles, which are oriented in an envelope conformation (ESI Fig. S1†).

**Table 2:** Selected bond lengths (Å) and angles (°) for In[Me_2_NCH(Me)CH_2_S]_2_Cl (1)

Bond length (Å)		Bond angle (°)	
In(1)–S(1)	2.426(2)	N(1)–In(1)–N(2)	171.6(2)
In(1)–S(2)	2.411(2)	N(1)–In(1)–S(1)	82.86(17)
In(1)–N(1)	2.390(7)	N(1)–In(1)–S(2)	92.40(17)
In(1)–N(2)	2.6115(6)	N(2)–In(1)–S(1)	94.68(18)
		N(2)–In(1)–S(2)	83.64(17)
		N(1)–In(1)–Cl(1)	92.83(18)
		S(1)–In(1)–Cl(1)	109.32(9)

The TG analysis of 1 revealed that it undergoes a single step prompt decomposition at ∼230 °C (ESI Fig. S2†). The weight loss observed (54.8%) corresponds to the formation of In_2_S_3_ from 2 moles of 1 (calculated weight loss 55.9%). The insights from TG analysis indicate that 1 can act as an efficient SSP for the synthesis of indium sulfide material. Accordingly, 1 was thermolyzed in OAm at 150 °C by the hot injection method. Subsequently, the nanostructures were allowed to grow for 1 min to prepare the colloidal indium sulfide NSs. The solvent OAm was primarily chosen due to the fact that it can act as a strong capping agent. OAm, being a primary amine, interacts with the molecular precursor, thereby behaving as a nucleation initiator and accelerates its decomposition.^29^ Moreover, it promotes the formation of monodisperse particles by separating the nucleation and growth steps.^30^

The phase purity and crystal structure of the synthesized nanostructures was evaluated by pXRD analysis (Fig. 1b). The prominent Bragg's reflections at 2*θ* = 20.0, 27.1, 32.0, and 48.0° in the pXRD pattern of the nanostructures can be well indexed to the reflections originating from the (211), (311), (400), and (440) planes of cubic In_2_S_3_ material (JCPDS 65-5526). The broadening of the peaks is typical for nanosized materials. The absence of any reflections from any other composition or phases of indium sulfide in the XRD pattern of the nanostructures confirms the phase purity of the material. It is worth noting that few reflections are missing in the pXRD pattern of the as-prepared In_2_S_3_ NSs, which is probably due to the restricted growth of the crystallite along those directions. Similar observations are quite common in case of materials falling under nanoregime.^16^ The average crystallite size, as calculated from the Scherrer equation,^31^ was found to be ∼12 nm. The findings of pXRD were well corroborated by EDS analysis, which confirms a 2 : 3 atomic percentage ratio of In : S (39.4 : 60.6) (ESI Fig. S3†). Raman spectroscopy is an important tool to investigate the phase purity of any material as it is difficult to differentiate between the impurities incorporated in the desired material from any other phase or composition by pXRD alone. Raman spectroscopy gives direct insights into the vibrational properties of nanocrystalline materials. The Raman spectrum of the material (Fig. 1c) illustrates characteristics peaks at 146, 195, 147, 294, and 320 cm^−1^ corresponding to the E_g_(2), F_2g_(1), B_2g_(2), E_g_(4), and E_g_(5) vibration modes, respectively. These observations are well supported by the previously reported values for In_2_S_3_.^32^ Further, to investigate the valence state and the chemical composition of the as-prepared sample, high-resolution XPS was carried out (ESI Fig. S4†). Prominent peaks at 161.7 and 163 eV (corresponding to S2p_3/2_ and S2p_1/2_, respectively) and 444.5 and 452 eV (corresponding to In 3d_5/2_ and 3d_3/2_, respectively) with spin orbit separation of ∼1.3 and 7.5 eV, respectively, suggest the existence of only S^2−^ and In^3+^ in the final product (Fig. 1d and e), which is in agreement with the previous reports.^33^ Furthermore, the XPS study also indicates that no observable surface oxidation has taken place in the material.

The morphology analysis of the material was carried out by the SEM and TEM techniques. The SEM image presented in Fig. 1f reveals the presence of ultrathin NSs oriented in a random fashion. It was also evident that the NSs are nearly transparent, and a fair idea about the average thickness of the NSs from the SEM study can be perceived, which was found to be ∼2 nm. A discrete NS with ultrathin morphology, as observed in the bright field TEM image (Fig. 1g), nicely corroborates the findings of the SEM analysis, while the HRTEM data reveals clear lattice fringes with a d-spacing value of 2.7 Å, which can be indexed to the (400) planes of cubic In_2_S_3_ (Fig. 1h). The highly nanocrystalline nature of the material can also be understood from the presence of concentric diffused rings in the SAED pattern (Fig. 1i). To have better clarity about the thickness of the NSs, semi-contact mode AFM was performed. The AFM image presented in Fig. 1j shows stacked NSs along with some discrete NSs. A line scan across the area featuring discrete NSs (area 1 (A1) and area 2 (A2)) was performed. The corresponding height-profile plot, as presented in Fig. 1k, reveals that the average thickness of the NSs is ∼1.76 nm. The TEM image (Fig. 1g) further supports the findings of the SEM and AFM studies by divulging the presence of randomly-oriented nearly-transparent ultrathin NSs. Furthermore, to confirm whether the constituent elements are homogeneously distributed in the material, 2D elemental mapping was performed. The mapping data (ESI Fig. S5†) gives a fine validation of the homogeneous distribution of the constituent atoms (In and S) in the resulting NSs.

**Fig. 2:**
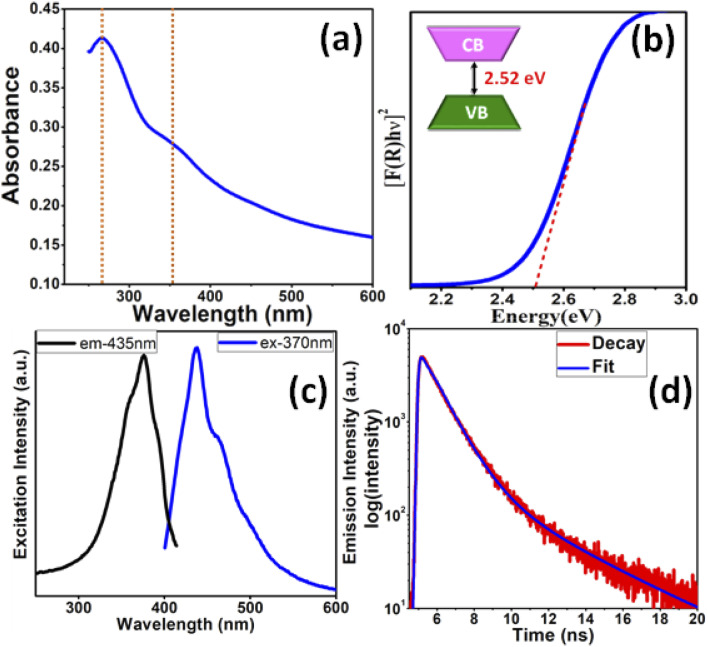
(a) UV-vis absorption spectra, (b) plots of [*F*(*R*)*hν*]^2^*vs.* energy generated by the Kubelka–Munk transformation of solid-state diffuse reflectance data, (c) PL spectra, and (d) luminescence lifetime decay (excitation and emission wavelength: 370 and 435 nm, respectively) of In_2_S_3_ NSs.

The optical properties of the In_2_S_3_ NSs were evaluated by UV-vis and diffuse reflectance spectroscopy (DRS). The UV-vis absorption spectra depict two peaks at 267 and 360 nm (Fig. 2a), which correlate well with the In_2_S_3_ nanostructures prepared by other groups.^16,34^ The characteristic step-like shape of the bands can be attributed to the valence-to-conduction band transition.^35^ Since In_2_S_3_ is a direct band gap material, the same for the synthesized NSs was determined using a plot of the Kubelka–Munk function, *F*(*R*), as expressed in the following equation.[*F*(*R*)*hν*]^*n*^ = *A*(*hν* − *E*_g_)where *hν* is photon energy, *A* is a constant, *E*_g_ denotes the band gap, and *n* depends on the nature of optical transition. The direct band gap was calculated using Tauc's model (*n* = 2). The direct optical band gap of the ultrathin In_2_S_3_ NSs was found to be 2.52 eV (Fig. 2b). This value is further corroborated by calculating the band gap of the material from the Brus equation (ESI†) (∼2.51 eV) and also from the position of the UV-vis onset (∼2.58 eV). The direct band gap values observed in the present study are clearly blue-shifted as compared to the bulk counterpart (*E*_g_ = 2.2 eV for bulk In_2_S_3_),^36^ which is a clear indication of quantum confinement of the excitonic transition, as expected for ultrathin In_2_S_3_ NSs. It is important to note that both the average crystallite size and thickness of these NSs, as determined from the pXRD and microscopic studies, respectively, are much smaller than the calculated exciton Bohr radius of In_2_S_3_ (33.6 nm) (ESI†), which further supports the occurrence of quantum confinement along the thickness and explains the considerable blue shift of the absorption edge and band gap. Fig. 2c provides the excitation and emission spectra of the as-prepared In_2_S_3_ NSs. In the excitation spectra, an absorption peak centered at 370 nm with a small hump at 365 nm was observed. Upon close inspection of the absorption and excitation spectra, it was observed that the peak in the excitation spectra is at the absorption edge of the peak in the absorption spectra. This phenomenon is often witnessed in direct band gap semiconductors wherein the photon-excitation rate is more at the absorption edge.^35^ In the emission spectra, a strong emission peak at 435 nm can be clearly observed. In addition to this, a small hump at 443 nm can also be seen. The origin of the emission peak is attributed to the interband electron–hole recombination. The emission in the blue region in the In_2_S_3_ NSs proves its worth as a photoemissive material as the latter is defined as a material that emits photon/electron of desired wavelength (visible light in the present case), when a radiation of suitable frequency is incident on it (UV in the present case). The photoluminescence lifetime decay from the as-prepared In_2_S_3_ NSs near the emission peak wavelength following excitation at 370 nm is presented in Fig. 2d. The lifetime curve can be fit adequately with the biexponential decay function with decay lifetime ∼1 ns (82.17%) and 4 ns (17.83%). The longer lifetime is comparable to the excitonic luminescence decay lifetime previously reported for In_2_S_3_ nanoparticles.^34,35^

**Fig. 3:**
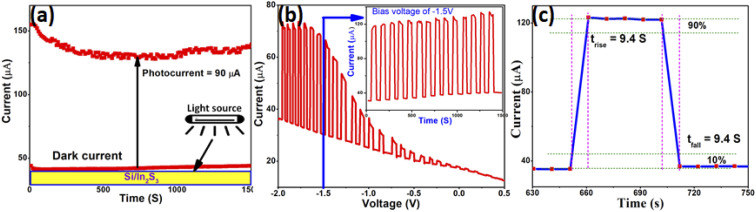
(a) Photostability and (b) *I*–*V* plot for In_2_S_3_ NSs conducted at a scan rate of 5 mV s^−1^. The inset shows the photoresponse under the illumination on–off condition by current *vs.* time plot at a constant potential of −1.5 V. (c) Expanded view of the middle (7th) cycle.

2D In_2_S_3_ nanomaterials are of paramount importance in optoelectronic and clean energy technologies. Ultrathin NSs, being a new morphology for In_2_S_3_, deserves an investigation into its photoresponsivity and photostability studies, which are the two most important criteria to select an absorber material for photovoltaic and photodetector applications. Photoresponsive materials have the ability to produce electron and thus, current, when a suitable light source is applied to them under a particular voltage. The *I*–*V* curve (ESI Fig. S6†) shows nonlinear characteristics, whereas the photostability studies of the material over a longer period of time under dark and illumination conditions resulted in a photocurrent (*I*_pc_ = *I*_light_ − *I*_dark_) of ∼90 μA (Fig. 3a). When the light was turned on, photoexcited electrons from the valence band reached the conduction band, resulting in increased photoconductivity. However, saturation is reached and no change in current is observed when the cell is exposed to light for a considerable amount of time. When the light was turned off, the “carrier generation process” abruptly stops, while the “carrier loss process” continues, resulting in a sudden decrease in the conductivity.^37^ Photoresponsivity (*R*) or photocurrent generation efficiency is the ratio of generated photocurrent and incident optical power (*R* = *I*_pc_/*PS*), which is expressed as the photocurrent (*I*_pc_) generated by the light power density (*P* = 200 μW cm^−2^) on the effective area of a photodetector (*S* = 0.785 cm^2^ in the present case). In the present case, the as-prepared In_2_S_3_ NSs exhibit high photoresponsivity of ∼570 mA W^−1^. Fig. 3b represents the *I*–*V* plot for In_2_S_3_ NSs conducted at a scan rate of 5 mV s^−1^, which shows the increasing magnitude of the photocurrent with increasing negative bias. It also represents rapid response of the In_2_S_3_ NSs to illumination, with peak-to-peak switching occurring in few seconds. To evaluate the response independent of a changing potential, constant voltage experiments were performed at −1.5 V. The graph is shown in Fig. 3b inset, which features switching curve with square wave response. The peak-to-peak photocurrents are seen to be quite stable. The electrode exhibits good switching for all 14 cycles, indicating good photosensitivity. The sudden decrease in conductivity in the dark condition is due to the loss of charge carriers. From the repeated cycles, the middle cycle has been chosen to calculate the rise time (*t*_rise_) (the time taken for the current to increase from 10% to 90% of the peak value) and decay or fall time (*t*_fall_) (the time taken for the current to decrease from 90% to 10% of the peak value). Both *t*_rise_ and *t*_fall_ for In_2_S_3_ nanostructure were found to be the same, *i.e.*, 9.4 s (Fig. 3c). The matching *t*_rise_ and *t*_fall_ is an indication of the involvement of less trap states or defects in the switching process.^38,39^ The appreciable performance of ultrathin In_2_S_3_ NSs is probably due to the unique morphology of the same by virtue of which it allows direct access to charge carriers with very high mobility.^40^ A comparative study of the photoresponse performance of the In_2_S_3_ NSs with some of the previous reports with different morphologies of In_2_S_3_ along with some other 2D materials (Table 3) reveals equivalent performance. These studies prove that ultrathin In_2_S_3_ NSs can be a valuable addition to the 2D semiconductor family.

**Table 3:** Photoresponse performance comparison of In_2_S_3_ nanostructures in the present work with previous reports

Nanomaterial	Synthetic route	Morphology	Band gap (eV)	Power intensity/light source	Bias voltage (V)	Photoresponsivity	Rise time	Decay (fall) time	Ref.
Si/In_2_S_3_ nanowires/Au/Ni composite photodetector	CVD	Nanowire	2.28	177 μW cm^−2^	5	7.35 × 10^4^ A W^−1^	6.5 ms	9.5 ms	41
In_2_S_3_ composite	Physical vapor epitaxy	Nanoflakes	—	405 nm	2	4812 A W^−1^	5.2 ms	4 ms	42
In_2_S_3_ thin film	Thermal evaporation & sulfurization	Nanoflakes	2.54	UV-vis	5	3.16 μA W	0.7 s	0.7 s	43
In_2_S_3_ thin film	Thermal evaporation	Islands	2.54	Visible	5	171 μA W^−1^	1.51 s	8.79 s	44
SnS	Thermolysis of SSP	Nanosheets	1.76	200 μW cm^−2^	1.5	38 mA W^−1^	17.04 s	8.89 s	37
InSe	Thermolysis of SSP	Ultrathin NSs	1.56	200 μW cm^−2^	1.5	19 mA W^−1^	18.6 s	8.8 s	18
In_2_Se_3_	Thermolysis of SSP	Nanoplatelets	1.79	200 μW cm^−2^	1.5	318 mA W^−1^	10 s	8.7 s	18
Sb_2_S_3_	Thermolysis of SSP	Nanorods	1.88	200 μW cm^−2^	1.5	312 mA W^−1^	9 s	8 s	45
Sb_2_S_3_	Polyol method	Nanoflowers	1.71	—	—	24.5 mA W^−1^	6 s	10 s	46
As-prepared In_2_S_3_	Thermolysis of SSP	Ultrathin NSs	2.52	200 μW cm^−2^	1.5	570 mA W^−1^	9.4 s	9.4 s	This work

## Conclusions

In summary, a new air- and moisture-stable indium complex with an aliphatic internally-functionalized hemilabile ligand was synthesized along with molecular structure determination. The complex acts as an efficient SSP for the preparation of phase-pure cubic In_2_S_3_ material. The AFM studies coupled with electron microscopic analysis indicate the formation of ultrathin NSs with an average thickness significantly smaller than the corresponding Bohr radius. The optical studies reflects quantum confinement in the as-prepared NSs. The measured band gap of ∼2.52 eV is in optimum range for these NSs to be potentially used as efficient absorber material in solar cells. They exhibit high photoresponsivity and photostability under alternating light and dark conditions. This new morphology of the 2D In_2_S_3_ material can further add new dimensions to diverse energy applications such as Li-ion batteries and thermoelectric devices. Moreover, since the SSP approach used to obtain the In_2_S_3_ NSs is simple, cost-effective, and has great potential for scale up, this study will also contribute toward the development of new SSPs for technologically important compound semiconductors with tunable properties.

## Conflicts of interest

The authors declare no conflicts of interest.

## Supplementary Material

RA-012-D2RA05000E-s001

RA-012-D2RA05000E-s002
