# Supplementary material for: Synthesis of photoresponsive and photoemissive ultrathin 2D nanosheets of In2S3 achieved through a new single source molecular precursor
Source: RSC Adv. 2022 Sep 27;12(42):27292–9. doi: 10.1039/d2ra05000e (PMC9513690; doi:10.1039/d2ra05000e)
Supplement: RA-012-D2RA05000E-s001 [file RA-012-D2RA05000E-s001.pdf]

Electronic Supplementary Material (ESI) for RSC Advances.
This journal is © The Royal Society of Chemistry 2022

Supplementary Information

Synthesis of Photo-responsive and Photoluminescent In_2S_3 ultrathin nanosheets achieved through a new single source molecular precursor

Gourab Karmakar,[a,b] Adish Tyagi,*[a,b] Alpa Y. Shah,[a] Liladhar B. Kumbhare,[a] A. P. Wadawale,[a] G. Kedarnath,*[a,b] Vishal Singh[c]

[a]*Chemistry Division, Bhabha Atomic Research Centre, Mumbai- 400 085 (India),*

[b]*Homi Bhabha National Institute, Anushaktinagar, Mumbai- 400 094 (India)*

[c]*Materials Science Division, Bhabha Atomic Research Centre, Mumbai- 400 085 (India).*

Email: tyagia@barc.gov.in, kedar@barc.gov.in

Determination of band gap from Brus equation

The Brus equation is given by[1]

$$E_{np} \approx E_{g(0)} + \frac{\hbar^2\pi^2}{2r^2}\left(\frac{1}{m_e^*} + \frac{1}{m_h^*}\right) - \frac{1.8e^2}{4\pi\epsilon R}$$

where E_{np} is the bandgap of the NSs, E_g is the band gap of bulk In_2S_3 (2.2 eV), ħ =h/2π is the reduced Planck constant, e is the electron charge, m_e* is the effective mass of electron, m_h* is the effective mass of hole, m_e* = m_h* (0.25 × 10^{-28} g), r is the average crystallite size of the NSs and ε is the dielectric constant (ε = 11). Putting all the values, the direct band gap of the ultrathin In_2S_3 NSs comes out to be ~2.51 eV.

Calculation of exciton Bohr radius of In_2S_3

The Bohr radius of the exciton in In_2S_3 can be calculated by the following equation[2]

$$r_B = \frac{\hbar^2\epsilon}{e^2}\left(\frac{1}{m_e} + \frac{1}{m_h}\right)$$

where ε is the dielectric constant (~11), $\hbar$ is the Planck constant and m_e and m_h are the electron and hole effective mass, respectively. $m_e = m_h = \mu$ = 0.25 x 10^{-28} g. From these data, Bohr radius of the exciton in In_2S_3 is calculated to be 33.6 nm.

Figure Captions:

Fig. S1. Molecular structure of $In[Me_2NCH(Me)CH_2S]_2Cl$ (**1**) showing envelop conformation of the ligands.

Fig. S2. Thermogravimetric analysis of $In[Me_2NCH(Me)CH_2S]_2Cl$ (**1**)

Fig. S3. EDS analysis of In_2S_3 NSs synthesized from $In[Me_2NCH(Me)CH_2S]_2Cl$ (**1**) at 150ºC for 1 minute.

Fig. S4. XPS survey scan of In_2S_3 NSs synthesized from $In[Me_2NCH(Me)CH_2S]_2Cl$ (**1**) at 150ºC for 1 minute.

Fig. S5. 2D elemental mapping of In_2S_3 NSs synthesized from $In[Me_2NCH(Me)CH_2S]_2Cl$ (**1**) at 150ºC for 1 minute.

Fig. S6. I-V characteristics of the In_2S_3 NSs synthesized from $In[Me_2NCH(Me)CH_2S]_2Cl$ (**1**) at 150ºC for 1 minute.

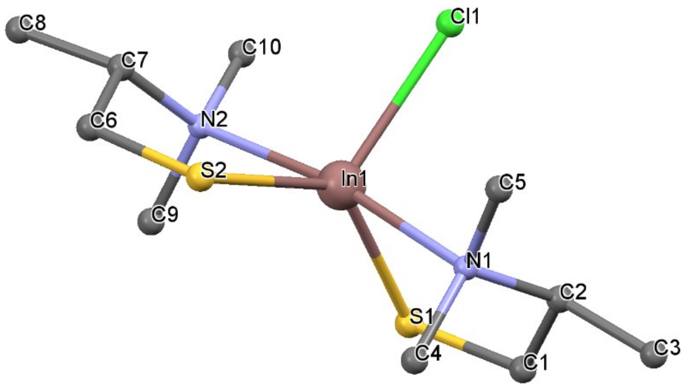

Fig. S1. Molecular structure of $In[Me_2NCH(Me)CH_2S]_2Cl$ (**1**) showing envelop conformation of the ligands.

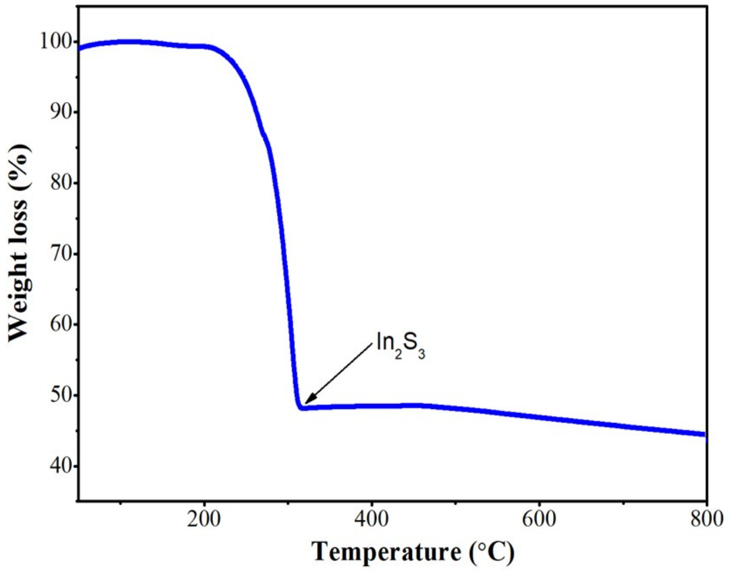

Fig. S2. Thermogravimetric analysis of In[Me_2NCH(Me)CH_2S]$_2$Cl (**1**)

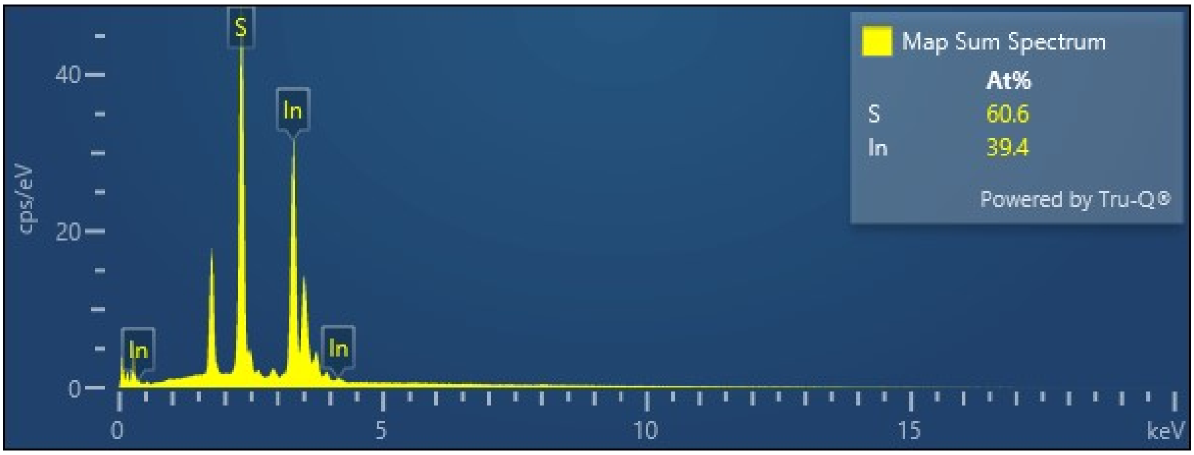

Fig. S3. EDS analysis of In_2S_3 NSs synthesized from In[Me_2NCH(Me)CH_2S]$_2$Cl (**1**) at 150ºC for 1 minute.

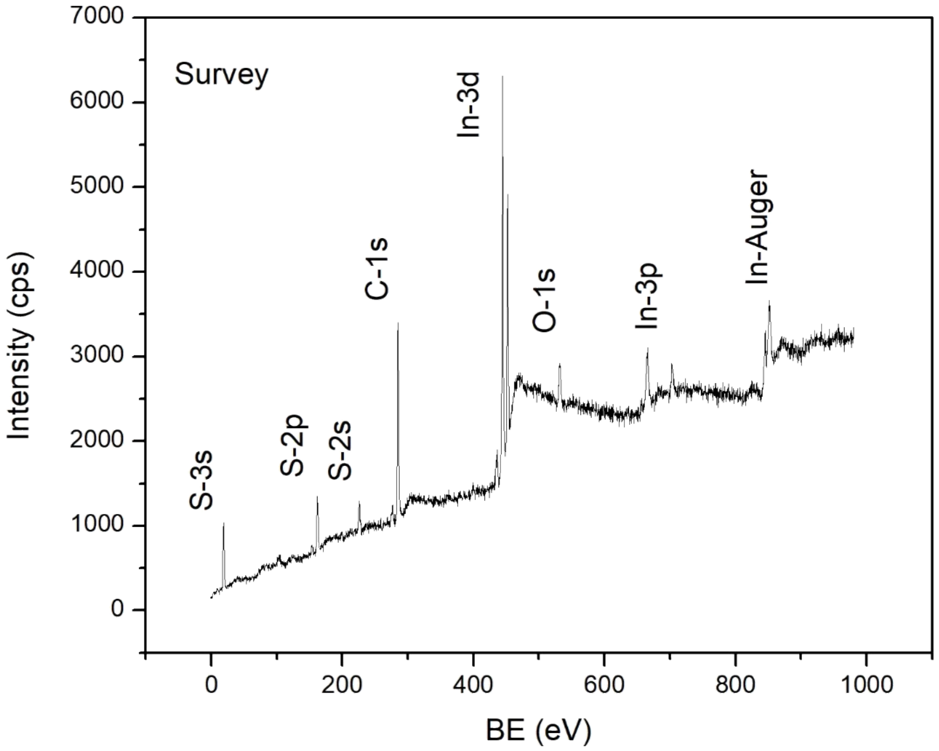

Fig. S4. XPS survey scan of In_2S_3 NSs synthesized from In[Me_2NCH(Me)CH_2S]$_2$Cl (**1**) at 150°C for 1 minute.

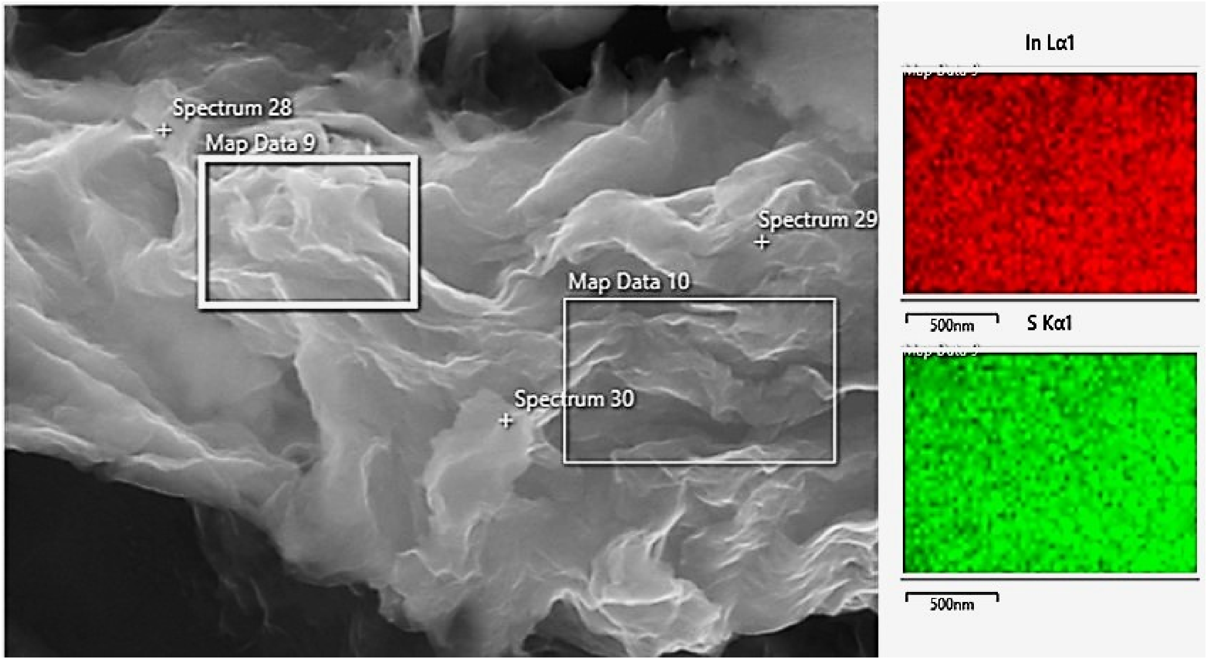

Fig. S5. 2D elemental mapping of In_2S_3 NSs synthesized from In[Me_2NCH(Me)CH_2S]$_2$Cl (**1**) at 150°C for 1 minute.

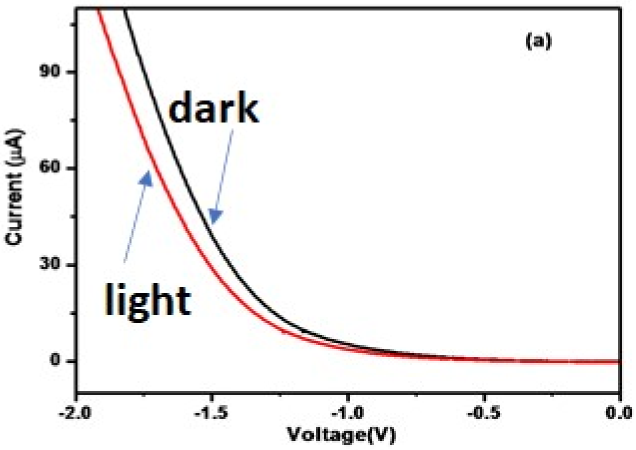

Fig. S6. I-V characteristics of the In_2S_3 NSs synthesized from $In[Me_2NCH(Me)CH_2S]_2Cl$ (**1**) at 150°C for 1 minute.

References:

1. R. Li, L. Tang, Q. Zhao, T. H. Ly, K. S. Teng, Y. Li, Y. Hu, C. Shu and S. P. Lau, *Nanoscale Res. Lett.*, 2019, **14**, 161.
2. W. Chen, J.-O. Bovin, A. G. Joly, S. Wang, F. Su and G. Li, *J. Phys. Chem. B*, 2004, **108**, 11927-11934.
